# Mesenchymal Stromal Cells from Different Parts of Umbilical Cord: Approach to Comparison & Characteristics

**DOI:** 10.1007/s12015-021-10157-3

**Published:** 2021-04-15

**Authors:** Ekaterina Semenova, Mariusz P Grudniak, Eugeniusz K Machaj, Katarzyna Bocian, Magdalena Chroscinska-Krawczyk, Marzena Trochonowicz, Igor M Stepaniec, Magdalena Murzyn, Karolina E Zagorska, Dariusz Boruczkowski, Tomasz J Kolanowski, Tomasz Oldak, Natalia Rozwadowska

**Affiliations:** 1 Research and Development Department, Polish Stem Cell Bank, FamiCord Group, Ul. Jana Pawla II 29, 00-867 Warsaw, Poland; 2 Faculty of Biology, Department of Immunology, University of Warsaw, Warsaw, Poland; 3 Clinic of Paediatric Neurology, III Faculty of Paediatrics, Medical University of Lublin, Lublin, Poland; 4 Institute of Human Genetics, Polish Academy of Sciences, Poznan, Poland

**Keywords:** Mesenchymal stromal cells (MSCs), Umbilical cord (UC), Wharton’s Jelly (WJ), MSCs from perivascular region (PRV), MSCs from UC membrane (UCM)

## Abstract

Mesenchymal stromal/stem cells (MSCs) are a unique population of cells that play an important role in the regeneration potential of the body. MSCs exhibit a characteristic phenotype and are capable of modulating the immune response. MSCs can be isolated from various tissues such as: bone marrow, adipose tissue, placenta, umbilical cord and others. The umbilical cord as a source of MSCs, has strong advantages, such as no-risk procedure of tissue retrieval after birth and easiness of the MSCs isolation. As the umbilical cord (UC) is a complex organ and we decided to evaluate, whether the cells derived from different regions of umbilical cord show similar or distinct properties. In this study we characterized and compared MSCs from three regions of the umbilical cord: Wharton’s Jelly (WJ), the perivascular space (PRV) and the umbilical membrane (UCM). The analysis was carried out in terms of morphology, phenotype, immunomodulation potential and secretome. Based on the obtained results, we were able to conclude, that MSCs derived from distinct UC regions differ in their properties. According to our result WJ-MSCs have high and stabile proliferation potential and phenotype, when compare with other MSCs and can be treated as a preferable source of cells for medical application.

**Graphical abstract:**

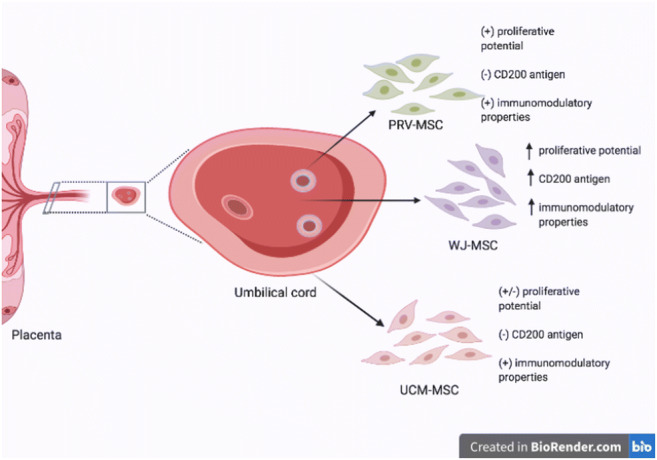

## Introduction

Mesenchymal stromal/stem cells (MSCs) can be isolated from different tissues or organs: bone marrow, adipose tissue, placenta, umbilical cord, amniotic fluid, liver, dental pulp, mobilized peripheral blood and others, but the most frequently used sources of MSCs remain bone marrow, adipose tissue. Unlike perinatal organs, adult tissues like bone marrow or adipose tissue have a number of limitations, such as: invasive acquisition procedure, higher risk of infectious diseases transmission, donor’s age, and limited proliferative potential of MSCs [1–4].

**Fig. 1:**
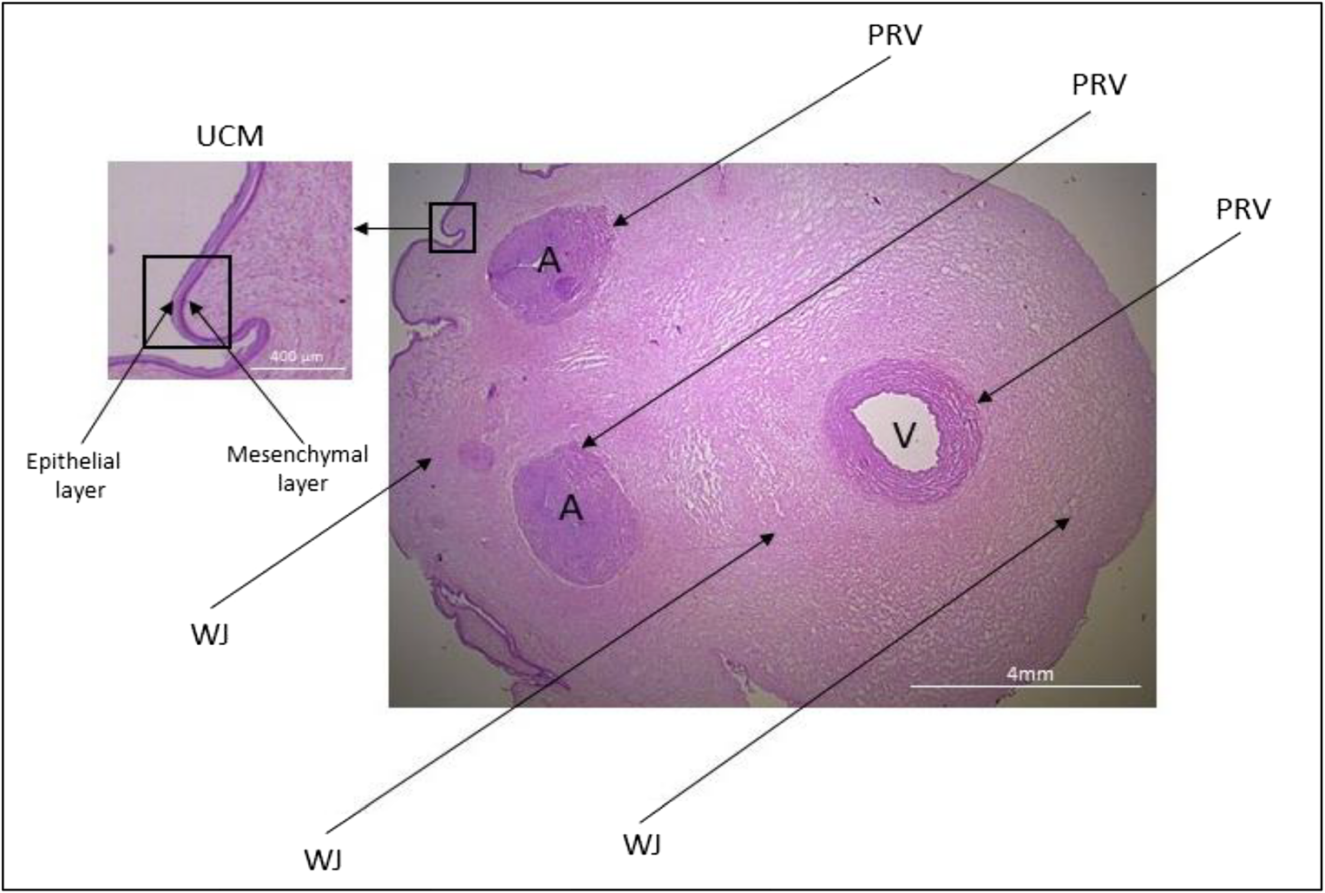
Cross-section of fresh umbilical cord fragment, Masson staining. Showing the compartments from which cells have been isolated: UCM- the umbilical cord membrane (epithelial and mesenchymal layer), WJ- Wharton’s jelly, PRV- the perivascular region, other abbrev.: A – arteries, V – vein

Umbilical cord (UC) is a perinatal organ connecting placenta and fetus, so as to facilitate the exchange of nutrition and gases (oxygen and carbon dioxide). Cross-section of umbilical cord (Fig. 1) demonstrates, that UC is composed of various anatomical parts: amniotic membrane (UCM) – umbilical cord membrane, which contain two layers: epithelial and mesenchymal, perivascular region (PRV) surrounding and protecting blood vessels, and the central part of UC constituted by Wharton’s Jelly (WJ) - a gelatinous substance rich in glycosaminoglycans, such as hyaluronic acid, chondroitin sulfate, which give that organ elasticity. Collection of UC is non-invasive and not associated with ethical issues, furthermore it is a good source to obtain considerable number of MSCs. These cells are characterized by a set of positive (CD73, CD90, CD105) and negative (CD45, CD14, CD34, CD19, HLA-DR) markers, adhesion to plastic surface and the capability of differentiation to other cell types, such as adipocytes, chondrocytes and osteoblasts [5, 6] . The unique multipotency, immunomodulation capabilities with low or none immunogenicity, due to the low expression of HLA class II proteins define MSC’s as potential source of therapeutic agents for regenerative medicine. The immunomodulatory features allow to use them in allo- and autogenic configuration [7, 8]. Another important aspect of clinical use is the secretion of various growth factors and cytokines, such as G-CSF, HGF, PDGFAA, TGF-β, IL-6, IL-8 and others, which play important roles not only in immunomodulation but also in promotion of cell proliferation, differentiation, growth and tissue repair [9–11].

Currently, MSCs are tested in more than 1000 clinical trials in plethora of diseases and conditions (Mesenchymal stromal/stem cells search at www.clinicaltrials.gov). Use of autologous and allogeneic MSCs in study of cardiovascular diseases treatment was resulted in cardiac functions improvement, MSCs were well tolerated and no serious adverse effects were observed when infused intramyocardially/ transendocardially into patients [12–14]. MSCs application to treat multiple sclerosis resulted in enhancing myelin regeneration and secretion of various cytokines/chemokines/growth factors, such as superoxide dismutase-3 in experimental models and clinical applications [15–19]. Developing new strategies in neurodegenerative diseases like Parkinson’s and Alzheimer’s disease allowed to modulate inflammatory environment, increasing neuron survival by paracrine effect of MSCs thanks to their ability to secrete potentially neuroprotective factors: EGF, VEGF, NT3, FGF-2, HGF, BDNF [20–23] . Several ongoing clinical trials to treat bone and cartilage diseases are being conducted to determine the usefulness of MSCs, including ability to differentiate to chondrocytes and to secrete growth factors. MSCs application in knee cartilage injury allowed to repair cartilage defect in any knee compartment using WJ-MSC–embedded scaffolds [24, 25].

Although, UC appears to be a relatively homogeneous tissue, there are reports, that the MSCs present in UC are different, depending on the region of isolation [26]. In 2005 Sarugaser’s group defined cells from perivascular UC area as MSCs progenitors [27] . Troyer et al. demonstrated the unique capabilities of MSCs from WJ [28], while Kita et al. isolated and characterized cells from umbilical cord membrane [29]. Nevertheless, the complex study, including the comparison of different regions from each UC in terms of gene and cytokine profiles have not been performed. The aim of our study was to deepen our knowledge about cells derived from three regions of UC: WJ, PRV and UCM. The study compared morphology, phenotypic profile, proliferation capabilities, differentiation possibilities, gene expression, cytokine profiles and senescence of MSCs isolated from three distinct UC regions.

## Materials and Methods

### Materials Collection

The umbilical cord (UC = 10) were collected by trained midwifes after caesarean sections as well as natural deliveries after mother’s informed consent, upon Ministry of Health approval. UC were washed in sterile 0,9% NaCl (FRESENIUS SE&CO, KGaA, Germany) and transported to the lab at 4 °C. After qualification the cords were processed 24 h after birth.

### Isolation and Expansion of MSCs

The cells were isolated from three different parts of umbilical cord (i) Wharton’s Jelly, (ii) perivascular region, and (iii) umbilical cord membrane region as it was shown in Fig. 1.

MSCs were isolated mechanically as described previously [8]. The main procedures were as follows: each part was gently separated and washed with sterile saline (0.9% solution of NaCl; FRESENIUS SE&CO. KGaA, Germany) supplemented with antibiotics/antimycotic solution, which contain 10,000 units/mL of penicillin, 10,000 μg/mL of streptomycin, and 25 μg/mL of Gibco Amphotericin B, as recommended for use at 10 mL/L (Antibiotic-Antimycotic (100x) catalog no. 15240096, Thermo Fisher Scientific, USA). Samples from each part were cut into small fragments (explants as small as 2–3 mm^3^), then washed twice with sterile saline, placed into 25 cm^2^ flasks (Thermo Fisher Scientific, USA) in complete StemMACS (TM) MSC Expansion Medium XF (catalog no. 130–104-182 Miltenyi Biotec, Germany) supplemented with antibiotics/antimycotic solution, which contain 10,000 units/mL of penicillin, 10,000 μg/mL of streptomycin, and 25 μg/mL of Gibco Amphotericin B, as recommended for use in cell culture applications at 10 mL/1 L (Antibiotic-Antimycotic (100x) catalog no. 15240096, Thermo Fisher Scientific, USA), followed by incubation in standard in vitro culture conditions (37 °C, 5% CO_2_). After 2 weeks of explants incubation, cells were collected and expanded in the conditions described above. Cells obtained from short-term (passage 3) and long-term in vitro culture (passage 8) were evaluated.

### Cell Proliferation Potential

Population Doubling Time (PDT) analysis was performed for assessment the proliferative potential of MSCs from UC (UC = 10). MSCs were seeded (10,000 cells/cm^2^) onto a T25 flasks until 90% confluence was reached. Adherent cells were detached with 0.25% Trypsin-EDTA (Thermo Fisher Scientific, USA), washed in PBS (Thermo Fisher Scientific, USA), centrifuged (300 x g, 10 min.) and resuspended in StemMACS (TM) MSC Expansion Medium XF. They were counted and PDT calculated according to the following formula:

$$ PDT=\frac{CT\times \mathit{\ln}2}{\mathit{\ln}\frac{N_f}{N_i}} $$

where: PDT – Population Doubling Time, CT – Culture Time in days, N_f_ – final Number of MSCs, N_i_ – initial Number of MSCs [30].

### Immunophenotypic Analysis

**Fig. 2:**
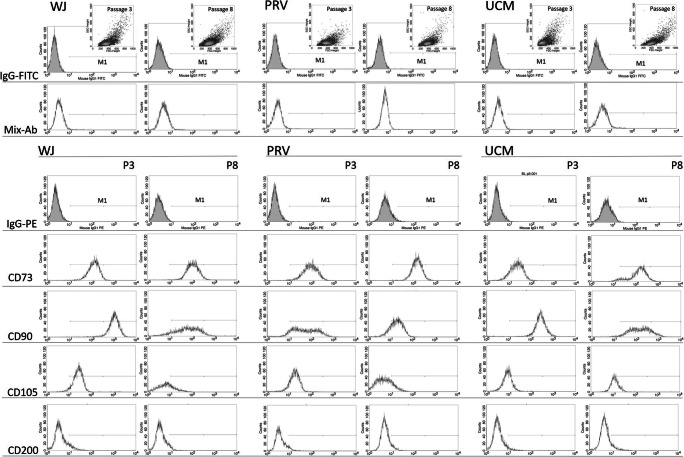
Representative flow cytometry result showing the expression of CD73, CD90, CD105, CD200 as well as the lack or minimal expression of Mix-Ab (CD14, CD19, CD34, CD45, HLA-DR) on the MSC derived from Wharton Jelly (WJ), perivascular region (PRV) and umbilical cord membrane (UCM). The expression of selected markers have been analyzed on cells shown on FSC/SSC dot plots. The gray histograms show the control labeling of cells with isotype antibodies: IgG-FITC, IgG-PE and the M1 area has been determined for positive cells (i.e. cells with expression of the observed markers)

To evaluate the surface antigen profile flow cytometry analysis was performed (UC = 10). Cells were incubated with phycoerythrin (PE)-conjugated monoclonal antibodies against human antigens: CD73 (catalog no. 550257, BD Pharmingen™, BD Bioscence USA), CD90 (catalog no. 561970, BD Pharmingen™, BD Bioscence USA), CD105 (catalog no. 560839, BD Pharmingen™, BD Bioscence USA), CD200 (catalog no. 552475, BD Pharmingen™, BD Bioscence USA), and fluorescein isothiocyanate (FITC)-conjugated monoclonal antibodies (later referred as the Mix-Ab) against: HLA-DR (catalog no. 555811, BD Pharmingen™, BD Bioscence USA), CD34 (catalog no. 345801, BD Pharmingen™, BD Bioscence USA), CD45 (catalog no. 345808, BD Pharmingen™, BD Bioscence USA), CD19 (catalog no. 345788, BD Pharmingen™, BD Bioscence USA), CD14 (catalog no. 345784, BD Pharmingen™, BD Bioscence USA) human antigens according to instruction manual. All antibodies including isotypic control were obtained from BD Bioscience, USA, IgG-PE (catalog no. 555749, BD Pharmingen™, BD Bioscence) and IgG-FITC (catalog no. 345815, BD Pharmingen™, BD Bioscence USA). Cells from passages 3 and 8 were selected as the data evaluation points. The cells were specifically analyzed by selective gating Fig. 2. The samples were analyzed with the FACS Calibur cytometer (BD Bioscience, USA) and CellQuest software (BD Bioscience, USA).

### Cell Viability

Viability of cells from passage 3 and passage 8 (UC = 10) were evaluated using the Accustain Solution, (Propidium Iodide – based) kit and ADAM-MC cell counter (Digital Bio, South Korea) according to the manufacturer’s protocol.

### Differentiation Assay

To evaluate MSCs differentiation potential, cells from the passage 3 (UC = 3) were used, according to the procedure described previously [7, 27, 31, 32]. In accordance with the recommendation, cells were seeded in density 5 × 10^3^/cm^2^ onto 24-wells plates (Corning, USA). Adipo-, osteo- and chondrogenic differentiation was initiated by StemPro Adipo-, Osteo- and Chondrogenesis Differentiation Medium (Thermo Fisher Scientific, USA), respectively. The culturing conditions were 37 °C, 5% CO_2_ in the air, and 90% of humidity. The medium was changed every 2–3 days. After 3 weeks, the culture media were removed and the cultured cells were fixed with 4% Formaldehyde solution (Sigma, USA). The fixed cells were washed with phosphate-buffered saline (PBS). Oil Red O (Sigma, USA) staining was used to evaluate the adipogenic potential. Cells differentiated into osteoblast were stained with Alizarin Red (Sigma, USA) to assess calcium deposits. In order to assess chondrogenesis, the cells were stained with Alcian Blue (Sigma, USA). All stained cells were analyzed with a Leica DM IL light microscope (Leica, Germany).

### Gene Expression Analyses

Purifying of total RNA extract from cells collected on P3 and P8 (UC = 3) was performed with RNeasy Plus Mini kit (Qiagen, Germany). Total RNA was treated with RNAse–free DNAse I kit (Qiagen, Germany). Reverse transcription was performed on using RevertAid First Strand cDNA Synthesis Kit (Thermo Fisher Scientific, USA). 500 ng of total RNA was used in this step. After reverse transcription, all cDNA samples were stored at −80 °C before analysis. qRT-PCR for the expression of genes: *RUNX, BGLAP, ADIPOQ, FABP4, COL10A1, COL2A1* and *C-MYC* was performed using SYBR Green Master Mix (Thermo Fisher Scientific). The primers used in our experiments were designed using NCBI primer BLAST and validated in silico with NETPrimer software. Designed primers were delivered by Genomed S.A. (Warsaw, Poland) (Table 1). Reactions were carried out in triplicate in a CFX 96 + C1000 cycler (Bio-rad, USA). PCR reaction was used under the following conditions: activation at 95 °C for 2 min., denaturation at 95 °C for 5 s., annealing at 60 °C for 30 s. for a total of 40 cycles. Immediately after last cycle, melting curve program were induced to ensure that only single, specific amplicons were produced. The results were analyzed according to the 2^-∆Ct^ and normalized to GAPDH and HPRT 1 using CFX Maestro™ Software 1.1 (Bio-rad, USA).

**Table 1:** Primers used for evaluation of differentiation genes activation

	Target gen	Primer	Sequence (5′➔3′)
Osteogenesis	*RUNX*	F	ACGGGGCACTGGGCTT
		R	GTGAGGGATGAAATGCTTGGG
	*BGLAP*	F	CCTCACACTCCTCGCCCTAT
		R	CTCTTCACTACCTCGCTGCC
Adipogenesis	*ADIPOQ*	F	GCAGTCTGTGGTTCTGATTCC
		R	CCCTTGAGTCGTGGTTTCCT
	*FABP4*	F	GAAAGGCGTCACTTCCACGA
		R	ATGCGAACTTCAGTCCAGGTC
Chondrogenesis	*COL10A1*	F	GCTGAACGATACCAAATGCCC
		R	CCTTGCTCTCCTCTTACTGCT
	*COL2A1*	F	TGGTGCTGCTGACGCT
		R	CTGTCCCTTTGGTCCTGGTT
Pluripotency genes	*SOX-2*	F	CGGAAAACCAAGACGCTCA
		R	GACCCCGCTCGCCAT
	*C-MYC*	F	CGCCTTCTCTCCGTCCTC
		R	TCTTCCTCATCTTCTTGTTCCTCC
reference genes	*GAPDH*	F	TGAAGGTCGGAGTCAACGG
		R	CTGGAAGATGGTGATGGGATTT
	*HPRT1*	F	CTGGCGTCGTGATTAGTGATGA
		R	GAGGGCTACAATGTGATGGC

### Secretome Analysis

The culture media were collected from MSCs (UC = 3) at passages 3 and 8, centrifuged for 10 min. at 10000 x g and supernatant was stored at −80 °C for the detection of proteins. A commercial magnetic bead panel kit (MILLIPLEX HCYTOMAG-60 K and MILLIPLEX MAP TGF-β1,2,3; Merck KGaA, Germany) was used for quantitative analysis of following chemokines (IL-8, MCP-1, MIP-1β, MIP-1α, RANTES, IP-10, Fractalkine), growth factors (TGF-β1, TGF-β2, TGF-β3, G-CSF, GM-CSF, EGF, PDGF AA, FGF2, Flt-3 L), pro-inflammatory cytokines (IFN-γ, IL-2, IL-1β, TNF-α, IL-3, IL-12, IL-17) and anti-inflammatory cytokines (IL-4, IL-5, IL-6, IL-10, IL-13, IFN-α). Sampling, processing and reagents preparation was performed according to the manufacturer’s protocol. Analysis was performed by Magpix instrument and with Belysa 1.0.19 software (both by Merck KGaA, Germany).

### Senescence-Associated β-Galactosidase Assay

To assess the activity of β-Galactosidase, the X-gal staining method was used. MSCs (UC = 3) derived from three different parts of UC (WJ, PRV, UCM) harvested at passage 3 and 8 were seeded into 6-well plates (BD Biosciences, USA) with a density of 10,000 cells/well and cultured in standard conditions until sub-confluent cultures displaying comparable cell density 30–40%. The cells were fixed with X-gal Fix buffer (0.1 M phosphate buffer (pH 7.3) supplemented with 5 mM EGTA (pH 7.3), 2 mM MgCl_2_ and 0.2% glutareldahyde) (Sigma, USA) at room temperature for 15 min. Then cells were washed with Wash buffer for X-gal staining (0.1 M, pH 7.3 phosphate buffer supplemented with 2 mM MgCl_2_), followed by the X-gal staining buffer addition and overnight cells incubation at 37 °C. Cells were washed with Wash buffer again and examined for the blue color presence with Leica DM IL LED microscope and images were captured with a connected digital camera (Leica, Germany) and analyzed with LAS V4.12 (Leica, Germany) software.

### Statistical Methods

Data were analyzed with Statistica software (StatSoft Polska). Statistical data are presented as means ± standard deviation for at least three replicates. Comparisons inside particular groups as well as between groups were done using ANOVA with significance set at a *p* value of less than 0.05.

## Results

### Isolation, Expansion and PDT Analysis of Cells

**Fig. 3:**
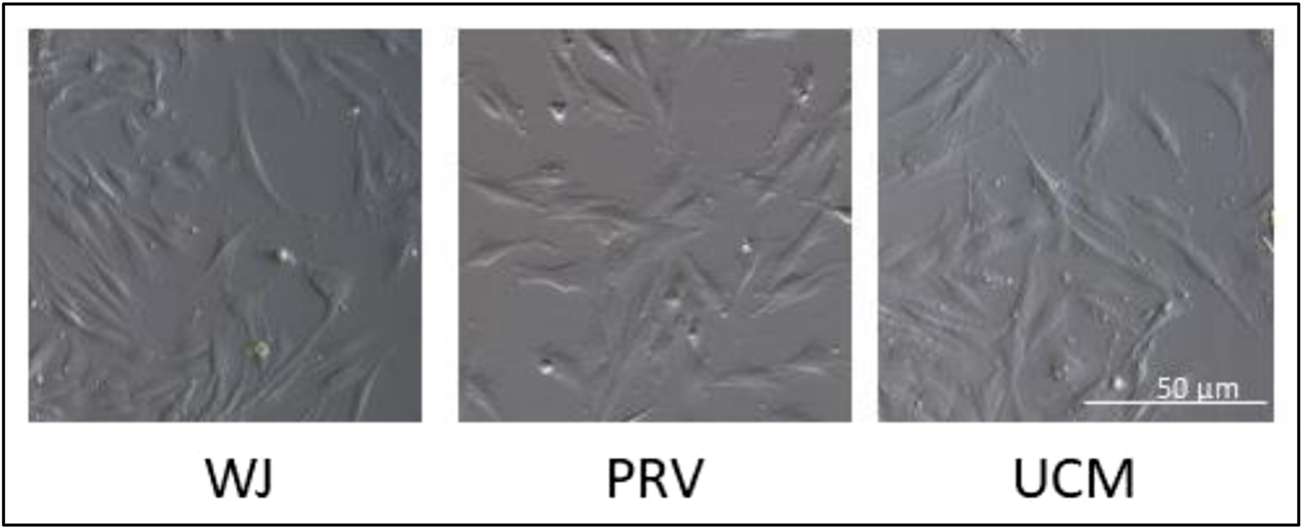
Representative morphology of cells derived from distinct umbilical cord regions: Wharton’s Jelly (WJ), the perivascular space (PRV) and the umbilical membrane (UCM). Cells grown as a typical spindle-shaped cells and formed a monolayer of adherent cells after of culture in StemMACS (TM) MSC Expansion Medium XF

MSCs were effectively isolated according to explants method described above from all three parts: Wharton Jelly (WJ), perivascular region (PRV) and umbilical cord membrane (UCM). The cells from all groups grew attached to plastic dishes and showed the characteristic MSC fibroblast-like shape (Fig. 3). The investigated MSCs demonstrated no significant differences in morphology.

**Fig. 4:**
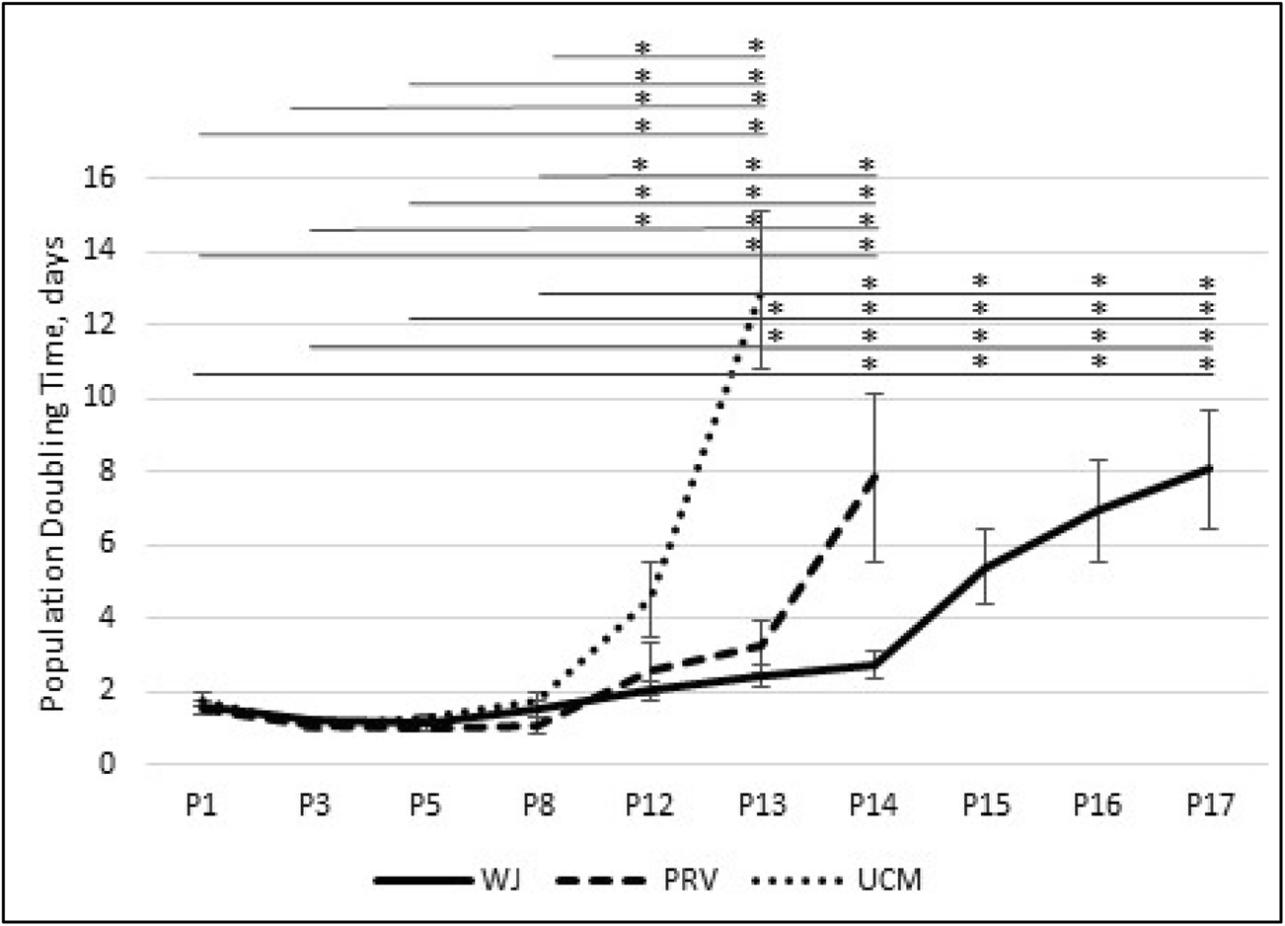
Growth kinetics of cells isolated from Wharton Jelly (WJ), perivascular region (PRV) and umbilical cord membrane (UCM) were evaluated using PDT (Population Doubling Time) analysis. Cultures were observed from passages 1,3,5,8,12–17. The horizontal lines above the graph indicate statistically significant differences between passages of the same compartment of umbilical cord (*p < 0,05)

The cells obtained from WJ, PRV, UCM showed similar growth characteristic from passages P1 to P8 and demonstrated congruous proliferation potential without statistically significant differences (Fig. 4). The mean PDT for all investigated lines was: WJ 1.35 ± 0.15, PRV 1.16 ± 0.12; UCM 1.47 ± 0.15. After passage 12 the cell division rates slowed in all types of tested cells. While cells from WJ and PRV during P12-P13 behaved similarly and maintained PDT within 2–4 days, the cells obtained from UCM demonstrated a rapid increase of PDT after passage 12, and subsequently lost the ability to proliferate at passage 13. The PRV showed consistent increase in PDT and the PRV cultures had to be terminated at passage 14. In the case of WJ cells, the period of PDT plateau was longer (P1-P14), with observed increase of PDT was after passage 14. WJ cells kept the proliferation capacity till the passage 17.

### Immunophenotypic Analysis

**Fig. 5:**
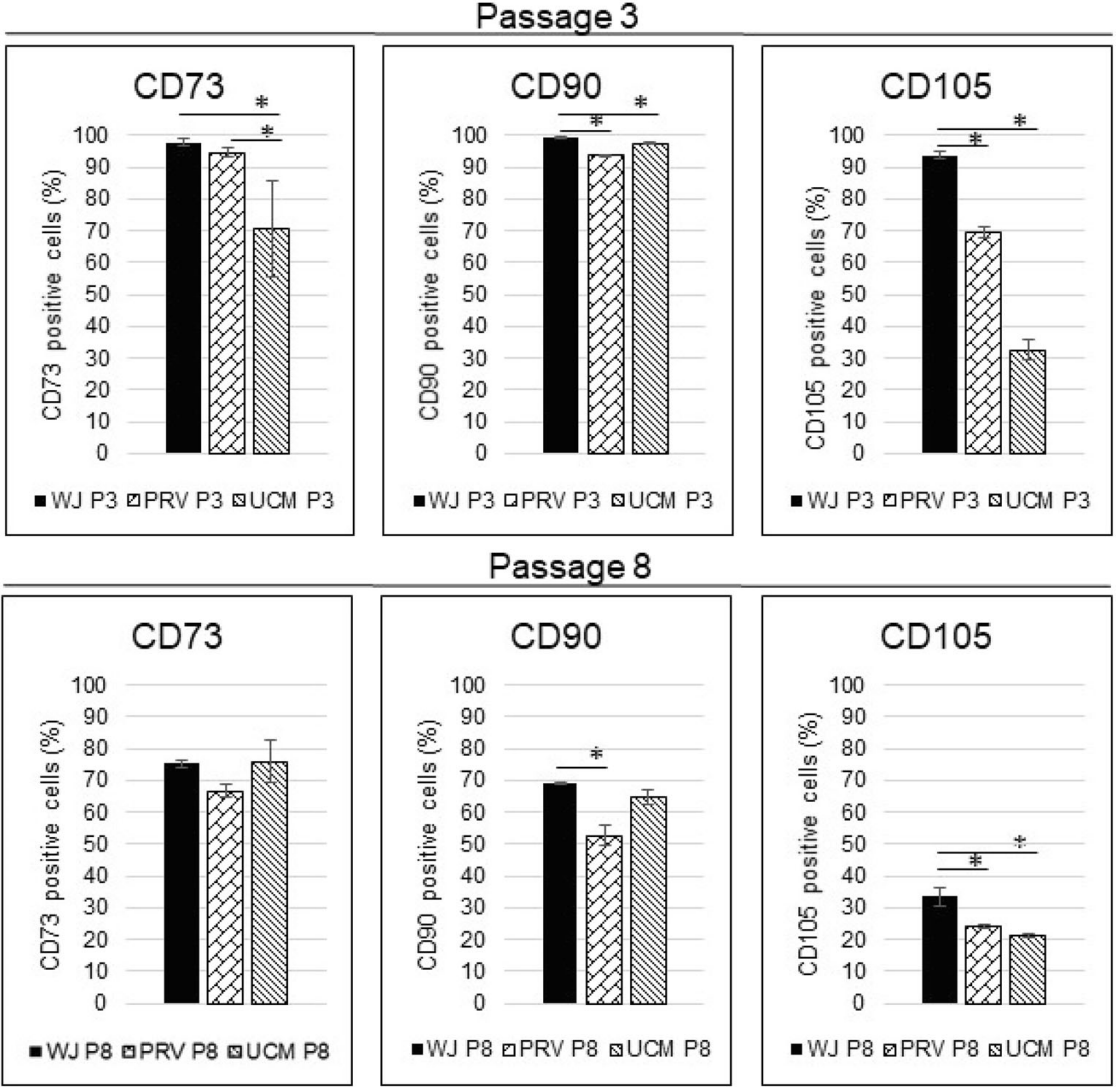
FACS analysis of characteristic phenotype of MSC cells derived from Wharton Jelly (WJ), the perivascular region (PRV) and the umbilical cord membrane (UCM). The expression of CD73, CD90, CD105 were identified on the MSCs with the lack or minimal expression of CD14, CD19, CD34, CD45, HLA-DR. The horizontal lines above the graph indicate statistically significant differences between cells from different parts of umbilical cord from passage 3 and 8. The flow cytometry analysis of the surface antigen profile was performed from 10 umbilical cords. (*p < 0,05)

All investigated cell populations derived from WJ, PRV and UCM from passage 3 and 8 included cells positive for CD73, CD90, CD105 and negative for HLA-DR, CD34, CD45, CD 19, CD14. In the case of negative marker cocktail, no significant differences were observed. However, cells from WJ and UCM from passage 3 showed significant differences in the percentages of CD73 positive cells (97.8 ± 1.3% and 70.8 ± 5.8% for WJ and UCM, respectively) (Fig. 5). The percentages of CD105 positive cells were significantly higher in cells isolated from WJ region in comparison to PRV and UCM (93.7 ± 1.3%; 69.7 ± 2.6%; 32.4 ± 9.8% for WJ, PRV, UCM, respectively). In the case of CD90, the percentage of positive CD90 cells at passage 3 was higher in WJ compared to PRV and UCM (99.3 ± 0.3%; 93.8 ± 0.2%; 97.6 ± 0.2% for WJ, PRV, UCM, respectively).

At the passage 8, the cells from all parts of UC showed decreased percentage of CD73^+^CD90^+^CD105^+^ cells, when compared to the cells assessed at passage 3. The exception were the cells from UCM region, where the percentage of CD73 positive cells increased by 7% in comparison to passage 3. Cells from P8 demonstrate lower percentage of CD73 positive cells in comparison to P3 but without statistically significant differences between regions (75.2 ± 1.8%; 66.5 ± 2.9%; 75.8 ± 8.9% for WJ, PRV, UCM, respectively). In the case of the CD90 marker, the percentage of CD90 positive cells from WJ was higher than those from PRV region (69 ± 0.7%; 52.7 ± 6%; 64.8 ± 3.3% for WJ, PRV, UCM, respectively). The percentage of CD105 positive cells was significantly higher in WJ (33.5 ± 9.1%) cells to compare with UCM (21.2 ± 2.5%) and PRV (24.1 ± 1.4%).

**Fig. 6:**
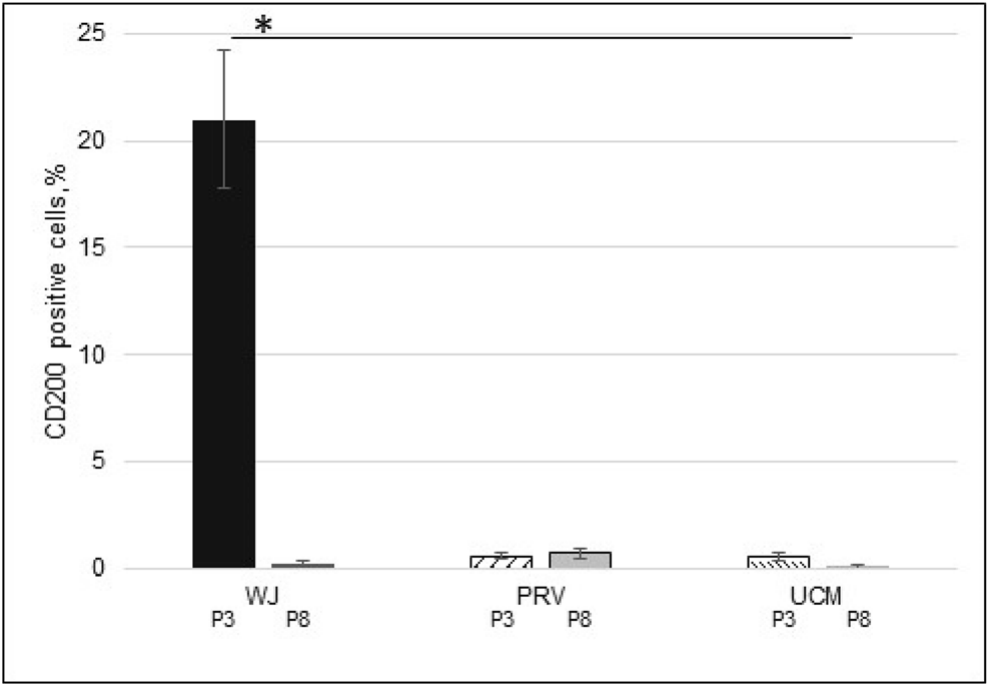
The flow cytometry analysis of CD200 expression in the cells from different regions: from Wharton jelly (WJ), perivascular region (PRV) and umbilical cord membrane (UCM) from passage 3 and 8. The horizontal line above the graph indicates statistically significant differences between WJ cells from passage 3 and the rest of MSCs. The flow cytometry analysis was performed from 10 umbilical cords (*p < 0,05)

The highest percentage of CD200 positive cells was found in cells from WJ in passage 3 (21%), then the percentage of CD200^+^ cells significantly decreased nearly to zero (0.2%) at the passage 8 (Fig. 6). On the other hand, CD200^+^ cells in PRV and UCM populations remain stable, yet very low population subsets at both P3 and P8 (about 0.6%).

### Cell Viability

Cells from investigated regions of UC demonstrated high viability, not less than 86%, even in those from passage 8 (data not shown). The highest percentage (97%) of viable cells was found in WJ cells from passage 3. A similar percentage of viable cells was found in all groups of cells from passage 8. Overall, there was a downward trend in the percentage of live cells throughout the culture period.

### In Vitro Differentiation

**Fig. 7:**
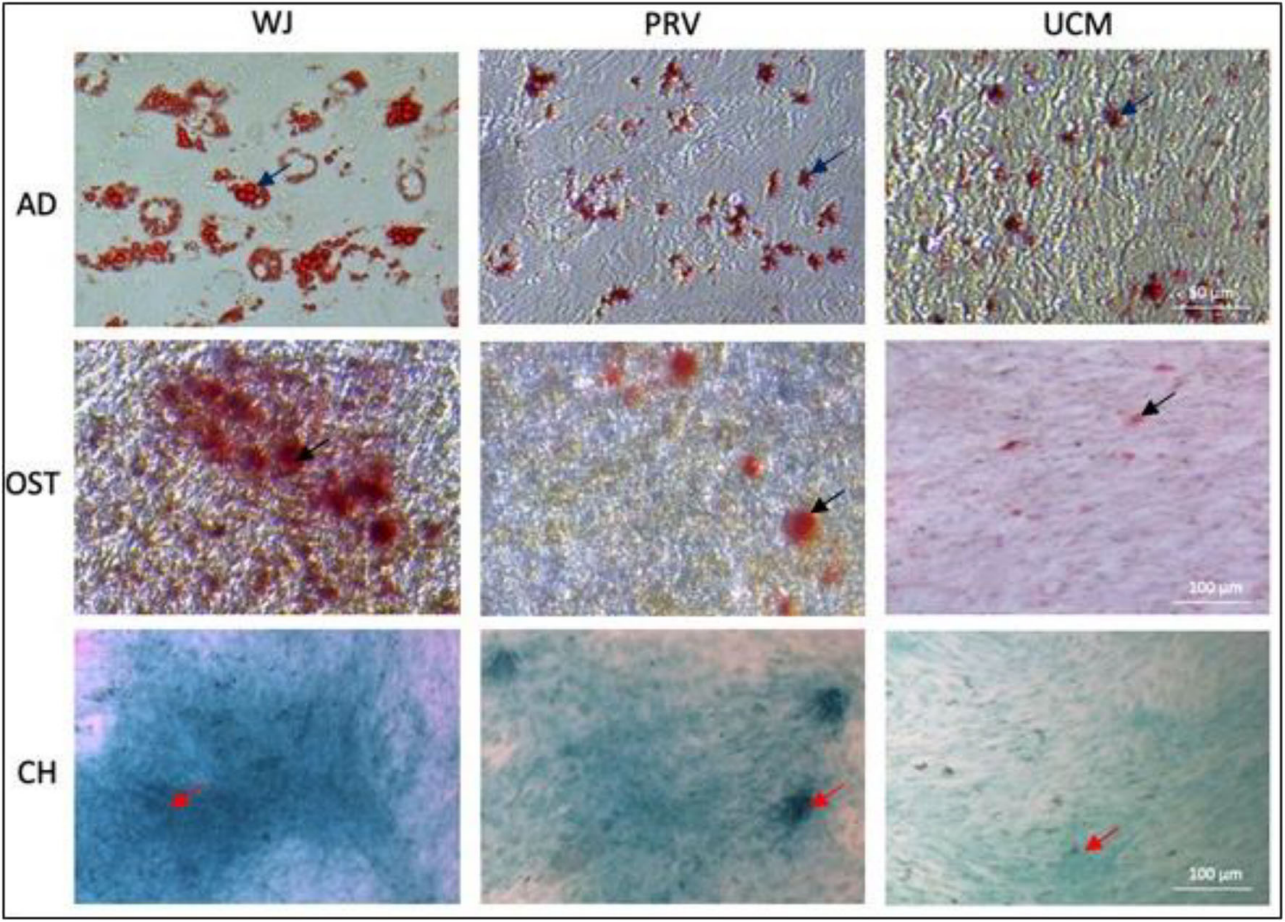
MSCs from WJ, PRV, UCM differentiate in adipocyte, osteocyte and chondrocyte lines. Representative differentiation of cells from passage 3 from Wharton jelly (WJ), perivascular region (PRV) and umbilical cord membrane (UCM) into adipogenic (AD), osteogenic (OST) and chondrogenic (CH) lineages. Adipogenic differentiation was examined by Oil Red staining, the differentiated culture demonstrates lipid vacuole formation typical for mature adipocytes (dark blue arrows) (scale bars, 50 μm); osteogenic differentiation was assessed by Alizarin Red staining of mineralized bone matrix (black arrows) (scale bars, 100 μm); chondrogenic differentiation was examined using Alcian Blue and blue color indicates cartilage extracellular matrix (red arrows) (scale bars, 100 μm)

Cells from WJ, PRV, UCM differentiated in adipocyte, osteocyte and chondrocyte lines (Fig. 7). Such type of differentiation test allowed only for qualitative assessment, however, the intensity of staining was evaluated globally, based on acquired images. Adipocyte colonies stained with Oil Red O were different in numbers: more stained colonies were observed in cells from WJ and PRV regions. In the case of osteocyte differentiation, cells from WJ region showed the highest intensity and number of staining substances. MSCs from the WJ and PRV treated with chondrocyte differentiation medium showed a higher number of stained cells than those from UCM.

### Gene Expression Results

**Fig. 8:**
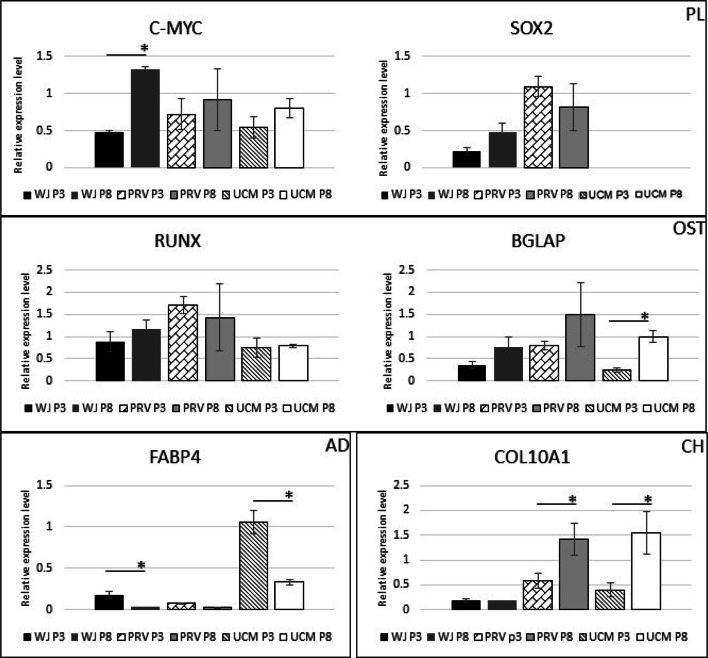
Comparison of gene expression profiles of cells from passage 3 (P3) and 8 (P8) from Wharton jelly (WJ), perivascular region (PRV) and umbilical cord membrane (UCM). **PL** - mRNA expression of pluripotency related genes *C-MYC*, *SOX2*; **OST** – mRNA expression of osteoblast related genes: *RUNX, BGLAP*; **AD** – mRNA expression level of adipocyte related gene: *FABP4*; **CH** – mRNA expression level of chondrocyte related gene: *COL10A1*. (*p < 0,05)

The expression of differentiation-related genes (osteogenic *RUNX, BGLAP*; adipogenic *FABP4, ADIPOQ*; chondrogenic *COL2A1, COL10A1*) and *C-MYC* was studied in order to analyze the differences in gene expression between MSCs from three different parts of umbilical cord (Fig. 8). *C-MYC* was expressed in all MSCs lines, the cells from WJ, PRV and UCM from passage 3 showed comparable level of mRNA expression. The highest level of *C-MYC* expression was shown by WJ-MSCs at passage 8. In general, an increase of *C-MYC* expression was observed from passages 3 to 8.

*FABP4* expression was observed in all studied cells, but the highest level was detected in the UCM cells. In the other cells *FABP4* expression level was very low. Interestingly, in all cells from passage 3, a tendency to greater expression was observed in comparison to the cells from passage 8.

Cells derived from short-term cultivation (P3) showed a comparatively low expression of terminal chondrocyte differentiation markers type X collagen (*COL10A1*). However, in the case of the cells from PRV and UCM, the expression of *COL10A1* increased 2–3 folds during the cultivation from passages 3 to 8. The typical adipocyte gene *ADIPOQ* and chondrocyte gene *COL2A1* expression was not detected in any MSCs. All cells expressed mRNA considered as osteogenic markers, such as *RUNX*, *BGLAP*. In the case of *RUNX* gene the highest expression was observed in PRV cells. Cells from WJ and UCM expressed *RUNX* at a comparable level and this parameter was stable during culture till P8. *BGLAP* was expressed by studied cells from all investigated parts of the umbilical cord. Increased *BGLAP* expression from P8 cells was observed in all cell groups. The highest level of *BGLAP* expression was observed in PRV cells in comparison to other studied groups.

### Secretome Analysis

MSCs secrete a wide range of growth factors, cytokines and chemokines. We have identified the presence of proteins, such as IL-1β, IL-6, IL-8, IL-13, MCP-1, EGF, Fractalkine, Flt-3 L, G-CSF, IFNα, IP-1, TGF-β1, TGF-β2, TNFα, PDGF AA (Table 2). The highest concentration of cytokines/chemokines for P3 cells concerned IL-6, TGF-β1, IL-8 and MCP-1. The statistically significant highest level of IL-6 level was observed in WJ-MSCs to compared with PRV- and UCM-derived (1070 ± 177.1, 539.8 ± 82.5, 612.3 ± 189 pg/ml, respectively) cells from passage 3. However, only cells from WJ showed stable level of IL-6 during in vitro culture. For PRV and UCM cells 3 times increased secretion of IL-6 in passage 8 was noticed (in the case of UCM cells and the difference in IL-6 level between passages 3 and 8 was statistically significant). TGF-β1 levels were comparable and constant in all types of P3 and P8 cells. The MCP-1 level was stable during in vitro culture of all three MSC-subtypes, but in the case of UCM cells the MCP-1 level was 5 times higher than observed WJ and PRV. The stable level of TGF-β2 was observed in PRV cells regardless of in vitro culture duration (139.2 pg/ml, 144.1 pg/ml, respectively), while in the case of WJ- and UCM-derived cells an increase was noticed in passage 8 to compare with cells from passage 3. In the case of cells from obtained from passage 3, lower level of IP-10 was observed in PRV cells in comparison to WJ and UCM cells. The level of IP-10 was increased in cells from each UC part in passage 8 to compare with passage 3. In the case of PDGF AA, the cells from WJ showed the 3 times increase in passage 8 to compare with passage 3; whereas the PRV-derived cells showed 7 times decrease in passage 8 in comparison to PRV cells from passage 3. The UCM cells from passage 8 presented large deviation in PDGF AA levels. The level of G-CSF in WJ and PRV cells remained unchanged during in vitro culture, however in the case of UCM-derived cells we were able to notice more than 20 times increase of expression of growth factor. The concentration of IL-1β, IL-13, TNFα, IFNα, fractalkine, FLT-3 L, EGF were not changed between passages, and comparable between UCM, PRV and WJ MSCs.

**Table 2:** The secretome of MSCs from Wharton jelly (WJ), perivascular region (PRV) and umbilical cord membrane (UCM). Marked concentrations represent the statistically significant differences (*p ≤ 0,05) *v.* passage 3. The rest of analyzed proteins concentrations remained unchanged without significant differences. The results of remaining analytes (FGF-2, GM-CSF, IFNγ, IL-10, IL-12p40, IL-17A, IL-2, IL-3, IL-4, IL-5, IL-7, MIP-1α, MIP-1β, TGF-β3) were at a very low level or outside the method resolution and were not used in this analysis

	WJ		PRV		UCM	
protein/	P3	P8	P3	P8	P3	P8
(pg/ml)						
IL-6	1070.1 ± 177.1	911.0 ± 225.2	539.8 ± 82.5	1900.3 ± 1439.1	612.3 ± 189.0	1690.3 ± 592.0*
TGF-β1	535.6 ± 194.7	398.0 ± 65.0	809.7 ± 151.4	606.5 ± 153.7	529.6 ± 172.4	607.5 ± 151
IL-8	367.0 ± 44.1	785.7 ± 170.1*	134.0 ± 25.9	415.3 ± 206.6*	211.9 ± 144.7	309.6 ± 266.1
MCP-1	121.8 ± 58.4	73.7 ± 11.4	93.9 ± 19.5	71.0 ± 7.8	505.9 ± 130.6	614.5 ± 364.4
TGF-β2	68.9 ± 20.8	165.8 ± 47.3*	139.2 ± 77.0	144.1 ± 19.8	67.2 ± 15.6	270.1 ± 17.6*
IP-10	16.6 ± 1.3	26.5 ± 3.7*	6.8 ± 2.0	24.6 ± 4.2*	14.3 ± 3.2	38.1 ± 12.0*
G-CSF	14.9 ± 5.3	10.8 ± 3.6	29.4 ± 4.8	14.6 ± 5.6	13.9 ± 5.1	945.9 ± 596*
PDGF AA	11.1 ± 2.6	35.6 ± 7.3*	56.6 ± 4.7	8.3 ± 3.1*	7.7 ± 1.9	15.6 ± 13.2
Fractalkine	27.2 ± 5.3	35.8 ± 5	22.0 ± 7.4	35.1 ± 4.9	20.5 ± 10.8	36.2 ± 14.3
IL-13	3.7 ± 1.1	3.4 ± 0.4	3.8 ± 1.0	3.3 ± 0.3	2.7 ± 0.1	3.9 ± 1.0
IFNα	8.4 ± 1.5	12.4 ± 2.3	9.4 ± 0.8	11.1 ± 3.4	10.3 ± 1.0	13.3 ± 5.6
Flt-3 L	5.6 ± 2.7	5.9 ± 2.2	4.8 ± 1.9	5.7 ± 3.4	5.8 ± 2.7	9.7 ± 4.0
EGF	4.4 ± 0.6	4.8 ± 0.4	4.2 ± 0.6	5.0 ± 0.5	4.0 ± 0.0	5.9 ± 1.0
TNFα	1.7 ± 0.3	1.4 ± 0.0	1.6 ± 0.0	1.6 ± 0.1	1.4 ± 0.3	1.8 ± 0.4
IL-1β	1.3 ± 0.1	1.3 ± 1.0	0.8 ± 0.2	1.1 ± 0.9	1.2 ± 0.0	3.5 ± 2.9

### Senescence-Associated β-Galactosidase Assay

**Fig. 9:**
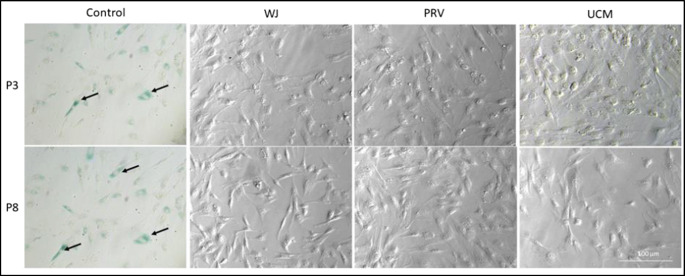
Representative β-Gal analysis in MSCs at P3 and P8 passages. Cells were analyzed under a light microscope. SA- β -gal activity was not noted in MSCs until P8. The figure is representative of cells grown from one donor for each cell type Wharton jelly (WJ), perivascular region (PRV) and umbilical cord membrane (UCM). As a control cells were used MSCs from UC isolated by standard method and treated by H_2_O_2_ to induce premature ageing representative blue cells, as markers of senescence, are indicated by black arrows

No senescent cells were found in the tested culture, even in P8 cells (Fig. 9). The absence of senescent cells is important to maintain cells self-renewal ability and differentiation potential.

## Discussion

The mesenchymal stromal cells from umbilical cord are a valuable tool for stem cell therapy and regenerative medicine [7, 27, 31, 32]. MSCs can be effectively isolated from different parts of UC: the perivascular region (PRV) surrounding the vessels, UC lining (UCM) and Wharton Jelly (WJ) as it was previously described [32–37]. Our study is one of the first focused on comprehensive characteristics of MSCs derived from different regions of human umbilical cord including phenotype, senescence, proliferation potential, expression and secretory profile. The aim of our study was to characterize cell population isolated from specific UC region to identify potential differences and determine their usage in future regenerative or/and immunomodulatory therapies. We investigated the effects of prolonged in vitro expansion (as long as passage 8) on phenotypic, proliferative, secretory, senescence and functional features of UC-derived MSCs [34, 35]. We also analysed selected gene expression profile in aspect MSCs origin and time of in vitro cultivation.

Xu et al. compared MSCs from different part of UC: whole UC, umbilical vessels, Wharton’s Jelly and amniotic membrane [35]. Their results demonstrated that MSCs from investigated regions possessed similar biological characteristics and properties to differentiate into osteoblasts, adipo- and chondrocytes and inhibited the proliferation of allogeneic T lymphocytes. Lim and Phat isolated MSCs from cord lining and confirmed that these cells had MSCs characteristics and differentiation abilities [33] . Subramanian et al. reported that cells from whole UC, amnion, subamnion, perivascular and WJ were typical “MSCs”, but, in fact, only MSCs from WJ characterized by high proliferation and differentiation potential and obtained populations were of higher purity than in other groups [36]. This data are with agreement with our findings that morphology and adherence properties are not different within all UC-derived MSCs population. We confirmed that cells isolated from different regions of UC grew attached to the plastic dish and in each case had fibroblast-like shape. However, only cells obtained from WJ cells fulfilled all ISCT minimum MSC phenotype criteria [5]. In our hands the phenotypes of cells derived from PRV and UCM were distinct, especially in the case of UCM cells, which showed significantly lower level of CD73^+^ and CD105^+^ cells. Our experiments confirmed that the cells from different parts of UC varied from each other not only by minimal criteria, but by more subtle parameters, such as proliferation potential, immunomodulation and protein secretion ability. According to our research, the main advantages of WJ-MSCs were that they showed high proliferation potential up to passage 14, with no signs of aging until passage 8 and ability to culture up to passage 17. In comparison PRV- and UCM-derived cells, maintaining morphology and phenotype only until to passage 8. We noticed a high percentage of CD200-positive cells from WJ analyzed on passages 3 in comparison to PRV and UCM cells [38, 39]. CD200 is involved in the immunoregulatory mechanism [40–43] and may provide a promising treatment strategy in neuroinflammatory diseases, like stroke, cerebral ischemia, Alzheimer’s disease, Parkinson’s disease, multiple sclerosis [44–49].

The presence of *C-MYC* expression in MSCs in all analyzed population also confirmed the fact that these cells possess a high proliferative capability [50]. Paula et al. demonstrated that growth factors from culturing media are able to increase expression of *C-MYC*, which leads to improvement of proliferative ability of the cells [51]. It should be added that *C-MYC* takes part in other biological processes, such as cell differentiation and apoptosis [52]. The expression of *C-MYC* in MSCs is not related to malignant transformation [52–55], and is present and indispensable in many other cell types. Melnik et al. confirmed that transplantation of generated MSCs with increased C*-MYC* expression did not result in tumor growth in any tissue in mice [55]. In fact, increased expression of *C-MYC* alone did not lead to MSCs transformation/malignant formation. Nair et al. confirmed that only the co-expression of *C-MYC* with *HER2 (ERBB2)* oncogene could result in increased risk of malignant transformation [53]. This finding was also confirmed by other scientific groups when *C-MYC* expression coexisted with loss/silencing of tumor suppressor genes led to MSCs transformation [54, 56]. The presence of lineage-specific genes at the mRNA level confirmed their physiological ability to differentiate into adipogenic, chondrogenic and osteogenic developmental lines. Higher expression of osteogenic-specific line genes (*BGLAP*, *RUNX*) probably indicates an ability to be prone towards osteogenesis. The lack of expression of *ADIPOQ* and *COL2A1* in undifferentiated MSCs was confirmed by other groups and can explained by the fact that these genes belong to genes expressed on more advanced stages of adipogenesis or chondrogenesis [57–61].

MSCs produce a wide range of cytokines, growth factors and chemokines. Due to secretory factors and direct cell-to-cell contact, MSCs exhibit their immunomodulatory activity [62–65]. IL-6 is important in the initial phase of the humoral immune response [66], together with TGF-β is involved part in balanced pro-inflammatory/anti-inflammatory reactions, directly inhibits Th17 response and stimulates the Treg population [66–71]. From the one side, presence of IL-6 with another pro-inflammatory cytokines promotes the induction of acute phase reaction, on the other it plays anti-inflammatory role, controlling the level of pro-inflammatory cytokines [68, 72, 73]. With this manuscript we join the others who proved, that MSCs secrete IL-6 at relatively high levels, which is suggested to be one of the key elements of MSCs immunomodulatory effects [74–78]. In our study, a high IL-6 level was presented in all cells, with the highest level was observed in p8 for PRV-MSCs.

The second important immunomodulatory factor is TGF-β, which was secreted by all analyzed MSCs populations. This growth factor is a key element for multiple vital functions of the cells including biological processes such as angiogenesis, immune response or endothelial-mesenchymal transition (EMT) [79–81]. As an immunomodulatory factor, TGF-β1 shows the potential to inhibit the proliferation of T-cells, B-cells, NK cells and other immune cells [63, 76, 80, 81]. TGF-β1 is able to stimulate and increase the Treg population [81, 82]. Interestingly, TGF-β participates in activation and recruitment of MSCs in tissue repair processes in cooperation with MCP-1, increasing MSCs migration to the injured area [83]. According to our analysis, stable level of TGF-β1 were presented by cells from passages 3 and 8, and suggests that stable and high level of TGF-β1 in cells is a second key element of immunomodulatory properties of MSCs. TGF-β2 together with TGF-β1 regulate crucial vital functions such as cell proliferation and differentiation. All studied cells on P3 showed relatively low levels of TGF-β2 due to the fact that this protein probably plays a minor role in the regulation of immunomodulation and other important cellular processes.

Our study confirmed the presence of MCP-1 in our investigated cells. In recent studies, the presence of MCP-1 (CCL-2) secreted by MSCs might be associated with immunomodulatory potential of these cells [84, 85]. The influence of MCP-1 on immunomodulation, including inhibition of immunoglobulin production by plasma cells and also influence on macrophage function, was emphasized [86, 87]. Additionally, MCP-1 able to promote MSCs cellular migration process [85, 88].

IL-8 produced by mesenchymal cells, belongs to proangiogenic, pro-migratory chemokine and participates in neutrophil recruitment [89]. The knockdown of IL-8 contributes to premature MSCs senescence, confirming that IL-8 is very important for MSCs functionality. The IL-8/CXCR2 signaling pathway is most probably essential for young cells to promote their growth and activity, while at the same time improving senescence in old cells is probably related to increased IL-8 levels in our P8 cells [90].

We did not detect IFN-γ, FGF-2, GM-CSF, IL-17A, IL-2, MIP-1α, TGF-β3 in any MSC supernatant and this observation is in agreement with previous published results [91, 92]. In the case of G-CSF, IFNα, PDGF AA our analysis shows their low concentrations in supernatants, which stays in line with other reports. Nevertheless, the presence of EGF, IL-1β, TNFα was confirmed in our study at low concentrations in supernatants, while IL-12, MIP-1β, IL-3, IL-4, IL-5, IL-10 were not noticed in supernatant, suggesting a difference in secretome composition [91, 92]. The differences between our results and results showed by others, could be explained probably by the differences in UC donor’s criteria, individual variation, health condition and age of each donor or may be sex of child, as previously mentioned [93–96].

WJ-MSC were used in many plethora of application. In neurology, WJ-MSCs are administered to patients with amyotrophic lateral sclerosis, multiple sclerosis, spinal cord injury as well acute and chronic stroke [88, 97, 98]. Research is also being conducted on WJ-MSC in neurological diseases in young patients with cerebral palsy, autism, traumatic brain damage and hypoxic-ischemic encephalopathy. WJ-MSCs were also used in hematology (aplastic anemia, graft-versus host disease (GVHD)), cardiology (chronic coronary disease, heart failure), immunology (systemic lupus erythematosus), musculoskeletal diseases (Duchene and Becker disease) and others (including rare diseases) [99–103] These clinical trials do not exceed phase II. Most of them have demonstrated safety in clinical use, but minority treated efficacy as a main endpoint. There were different doses and different routes of administration (including intravenous, local or intramuscular) [104]. Although being intensively investigated, the mechanism of action and the efficacy of MSC-based therapies remain the principal issue for MSCs therapy approval in many cases.

In conclusion, our data suggests that WJ-MSCs are definitely the most promising and stable source of homogeneous MSCs among UC tissues, and that the isolation of MSCs from UC may be limited to this area, which would potentially be beneficial in cell-based therapy. One should mention that the appropriate awareness should be applied during WJ-MSC preparation for clinical application to avoid contamination from other UC compartments as this could influence the quantities and qualities of obtained Advanced Therapy Medicinal Products.

## Data Availability

N/A

## Code Availability

N/A
